# COVID-19: Imbalanced Immune Responses and Potential Immunotherapies

**DOI:** 10.3389/fimmu.2020.607583

**Published:** 2021-01-29

**Authors:** Bing Xie, Jiancheng Zhang, Yuwen Li, Shiying Yuan, You Shang

**Affiliations:** ^1^ Department of Critical Care Medicine, Union Hospital, Tongji Medical College, Huazhong University of Science and Technology, Wuhan, China; ^2^ Hayward Genetics Center, Tulane University School of Medicine, New Orleans, LA, United States

**Keywords:** coronavirus disease 2019, severe acute respiratory syndrome coronavirus 2, pathogenesis, immunomodulation, IL-6R blockade, convalescent plasma, intravenous gamma globulin, thymosin alpha1

## Abstract

The ongoing pandemic coronavirus disease 2019 (COVID-19) caused by severe acute respiratory syndrome coronavirus 2 (SARS-CoV-2) is rapidly spreading and has resulted in grievous morbidity and mortality worldwide. Despite the high infectiousness of SARS-CoV-2, the majority of infected individuals are asymptomatic or have mild symptoms and could eventually recover as a result of their balanced immune function. On the contrary, immuno-compromised patients are prone to progress into severe or critical types underpinned by the entanglement of an overexuberant proinflammatory response and injured immune function. Therefore, well-coordinated innate and adaptive immune systems are pivotal to viral eradication and tissue repair. An in-depth understanding of the immunological processes underlying COVID-19 could facilitate rapidly identifying and choosing optimal immunotherapy for patients with severe SARS-CoV-2 infection. In this review, based on current immunological evidence, we describe potential immune mechanisms and discuss promising immunotherapies for COVID-19, including IL-6R blockades, convalescent plasma, intravenous gamma globulin, thymosin alpha1, corticosteroids, and type-I interferon, and recent advances in the development of COVID-19 vaccines.

## Introduction

The ongoing coronavirus disease 2019 (COVID-19) pandemic triggered by severe acute respiratory syndrome coronavirus 2 (SARS-CoV-2) has gripped the world in an unprecedented crisis, entailing issues with the economy, health care, and education. Millions of people worldwide have suffered from this highly contagious disease. The vast majority of infected patients had a good prognosis, but there were still some severe and critical cases and even deaths (1). Epidemiological studies show that the progression of pulmonary infiltrates and multiorgan failure was commonly concomitant with markedly elevated serum proinflammatory cytokines (i.e., interleukin (IL)-2R, IL-6, IL-10, IL-8, and tumor necrosis factor (TNF-α)) and other biomarkers of inflammation (i.e., procalcitonin, C-reactive protein (CRP), and ferritin) (2–4). Besides this, protracted and severe lymphopenia strongly correlates with adverse outcomes and fatality (4, 5). Together, these clinical observations indicate that, apart from direct viral toxicity, immune dysregulation serves as an independent and potent contributor to disease aggravation. Thus, a better understanding of the immunological mechanisms underlying the physiopathology in SARS-CoV-2–infected patients is necessary for identifying therapeutic targets and appropriate deployment of therapies. Large amounts of research have been conducted to evaluate immune-related therapies for COVID-19 in clinical applications or trials; however, a consensus on a specific therapeutic strategy has not yet been reached. In this review, we discuss the role of immune responses in the development of COVID-19 and summarize current clinical results of several promising immunomodulatory agents and vaccines for COVID-19 (**Table 1**).

**Table 1:** COVID-19: imbalanced immune responses and promising immunotherapies.

Intervention	Published data in COVID-19 as of July 19, 2020	Mechanism of action	Recommendations
IL-6R blockades: Tocilizumab Sarilumab	Observational studies with inconsistent results: Case series and retrospective cohort studies reported improved outcomes; one case report found no beneficial therapeutic effect	Monoclonal antibody against IL-6	Prudent use of IL-6 targeting therapies with antiviral agents in cases with early, rapid, continuous deterioration and clinical and laboratory proof of overactive inflammatory responses (i.e., rapidly aggravating state of breathing, shock, and elevations in IL-6 and CRP)
Convalescent plasma	Observational studies reported improvements in clinical outcomes; one randomized clinical trial with hopeful but not statistically significant results	Provides protective antibody against SARS-CoV-2	Application in patients displaying advanced disease course with severe viremia
IVIG	Observational studies report clinical improvements as an adjuvant agent	Provides passive immunity and anti-inflammatory effects	Application in COVID-19 as an adjuvant agent
Thymosin alpha1	Observational studies report therapeutic benefit in recovery of T cell numbers.	Polypeptide with immunoregulatory effects	Application as an adjuvant agent in COVID-19 cases, especially with lymphopenia
Corticosteroids	Observational studies found possible clinical benefit in ARDS, but there remains concern about prolonged virus shedding and hazard of high dosing; the recovery trial reports improvement in clinical outcomes	Inhibit inflammatory cells and dampen proinflammatory cytokines	Short-term and low-dose application in patients with respiratory disorders, simultaneously with close monitoring
IFNs	Type-I IFNs combined with other antiviral agents demonstrate clinical efficacy in one observational study and one phase II clinical trial	Suppress virus replication	Early administration with other antiviral agents preceding the peak of viremia or prophylactic treatment might confer protection

ARDS, acute respiratory distress syndrome; COVID-19, corona virus disease 2019; IFNs, interferons; IL-6R, interleukin 6 receptor; IVIG, intravenous gammaglobulin; SARS-CoV-2, severe acute respiratory syndrome coronavirus 2.

### COVID-19: SARS-CoV-2 Intrusion and Innate Immune Hyperactivation

SARS-CoV-2 is a large, enveloped, positive-sense, single-stranded RNA virus belonging to beta coronaviruses and shares 82% homology with the genomic sequence of SARS-CoV (6). The SARS-CoV-2 RNA genome covers approximately 30 kilobases and encodes four main structural proteins (the spike (S), envelope (E), membrane (M), and nucelocapsid (N) protein), 16 nonstructural proteins (Nsp1–16) and 7 accessory proteins (ORF3a, 3b, 6, 7a, 7b, 8, 9b, 9c, and 10) (7). Structural and nonstructural proteins synergistically sustain viral survival and infectious power, and accessory proteins regulate host responses to promote infection and pathogenesis. SARS-CoV-2 uses angiotensin-converting enzyme 2 (ACE2) as a portal for viral entry (8). ACE2 is ubiquitously expressed in almost all organs in human, among which type-2 alveolar cells possess an abundance of ACE2 (9), partially contributing to the commonly observed acute respiratory distress syndrome (ARDS) in critical COVID-19 cases. There is literature suggesting CD147 as a receptor for SARS-CoV-2 invasion (10); however, to what extent this pathway contributes to viral entry and replication requires further elucidation. Viral infection and replication in airway epithelial cells cause the damage to infected lung cells and vascular leakage as well as the release of inflammatory mediators (11). The released virus-associated damage-associated molecular patterns (DAMPs), involving intracellular contents from dying cells (12) as well as pathogen-associated molecular patterns (PAMPs), including viral RNA (13), are identified by multiple pattern-recognition receptors (PRRs) on innate immune cells, such as dendritic cells, monocytes, macrophages, neutrophils, and epithelial cells (14). Endosomal RNA PRRs involve toll-like receptors (TLR-)3 and 7, and cytosolic sensors include retinoic acid–inducible gene 1 (RIG-I) and melanoma differentiation–associated gene 5 (MDA5) (15). After the recognition of virus invasion, a series of intracellular inflammatory signaling pathways are then activated, and inflammatory products are then generated and secreted, among which, type-I IFN (IFN-I) is considered to play a central role in the first-line antiviral defense. Upon recognizing RNA ligands, the caspase activation and recruitment domain (CARD) is exposed and activates the mitochondrial antiviral adaptor protein (MAVS) to start downstream signaling. IFN regulatory factor (IRF)3 and 7 then undergo phosphorylation and translocate to the nucleus, inducing the expression of IFN-I genes and interferon-stimulated genes (ISGs) (16, 17). Moreover, TLR-3/7 activation leads to nuclear translocation of the NFκB and IRF3 (15). These transcription factors induce the expression of genes that encode cytokines (IL-1, IL-6, TNF-α, etc.) and chemokines (CXCL10, CXCL8, CXCL9, CCL2, etc.). The increase of multiple inflammatory cytokines and chemokines attracts a variety of innate immune cells as well as adaptive cells to the inflamed site for virus control (18).

Early sufficient production of IFN-I is paramount to the control of viral dissemination and effective viral clearance. However, low circulating levels of IFNs are regularly detected in severe COVID-19 patients (19) and seemingly related to illness severity (20). Notably, the contrasting observations of robust IFN responses, hallmarked by highly expressed ISGs, were detected in the bronchoalveolar lavage fluid (BALF) of some critically ill patients (21). Also, the succumbed patients displayed significantly elevated levels of plasma IFN-α and IFN-λ throughout the disease course, and these were highly correlated with viral load (22). The discrepancy of IFN dynamics between the inflamed lung and the circulatory system of severe and critical COVID-19 patients may reflect an incapability of viral control in very early stages of viral invasion, contributing to systemic compromised antiviral responses (low circulating IFN levels) or uncontrolled harmful IFN responses. Furthermore, the strong IFN responses in the lungs fail to restrain disease progression through suppressing viral replication, suggesting that SARS-CoV-2 might have evolved mechanisms to evade innate immune killing. Given the high homogeneity between SARS-CoV-2 and SARS-CoV, there are good theoretical reasons to believe that SARS-CoV-2 might exploit similar mechanisms to inhibit IFN-I response. SARS-CoV ORF3b is reported to interfere with IRF3 or MAVS to inhibit IFN induction (17). SARS-CoV is also found to inhibit the TNF receptor-associated factors (TRAF) 3 and 6, which are essential for the induction of IRF3/7 (23). Furthermore, it is reported that ORF9b of SARS-CoV-2 could inhibit MAVS activation, which is required for nuclear translocation of IRF3 (17). Importantly, SARS-CoV-2 Nsp1 is shown to bind to human 40S ribosomal subunits, thus blocking host mRNA translation involving that of IFN-I (24).

IL-6 is a prompt effector in response to acute infections, and it can be produced by extensive cells, including fibroblasts; endothelium cells; and immune cells, such as macrophages, monocytes, B lymphocytes, and T lymphocytes (25). Hyperactivated IL-6 might promote inflammation cascades. IL-6 functions partly through trans signaling *via* binding to soluble bound IL-6 receptor (sIL-6R), which is expressed by virtually all body cells (including stromal and epithelial cells) (26). The binding of IL-6 and widely expressed sIL-6R is likely to fuel the hyperinflammation and be partially responsible for severe ARDS and multiorgan failure in COVID-19. It is reported that IL-6 could serve as a predictive marker for the severity of COVID-19 (27). Evidence has shown that IL-6 levels in COVID-19 patients needing critical care continues to increase over time and are relatively more elevated in nonsurvivors than in survivors (28). However, circulating concentration of IL-6 and other inflammatory cytokines (e.g., IL-8, TNF-α, IFN-γ) in patients with severe and critical COVID-19 is far from the median levels of that in cytokine release syndrome, sepsis, and ARDS unrelated to COVID-19 (29). As such, the role of a cytokine storm wherein IL-6 is considered to be highly involved in the pathogenesis of COVID-19 warrants further exploration.

Abundant activated dendritic cells, activated mast cells, and neutrophils along with a significant neutrophil-to-lymphocyte (NLR) ratio were observed in the BALF of COVID-19 patients (21), which is supported by a study showing higher NLR in severe COVID-19 cases (30). Increased plasma levels of neutrophils and eosinophils were correlated with the severity of COVID-19 (22). In another single-cell RNA sequencing (scRNA-seq) analysis, severe COVID-19 patients with ARDS showed a pronounced increase of peripheral activated granulocytes. Altogether, these data indicate the pathogenic role of granulocytes in the development of COVID-19. In parallel, the dysregulation of monocytes and macrophages seems to be a driving contributor in the pathogenesis of severe SARS-CoV-2 infection although conflicting results exist. CCL2 and CCL7, as two potent chemokines for recruiting CC- chemokine receptor 2-positive (CCR2^+^) monocytes, were found in the BALF of severely infected COVID-19 patients (31). In addition, evidence indicates an activated phenotype of monocytes involved in the immunologic pathogenesis of COVID-19. Zhou et al. report a significantly higher proportion of inflammatory monocytes with a high expression of IL-6 and granulocyte–macrophage colony-stimulating factor (GM-CSF) in the peripheral blood of COVID-19 patients (32). Even in the recovery stage of COVID-19, Wen et al. observed a greater abundance of circulating CD14^+^IL-1β^+^ and IFN-activated monocytes in infected patients in comparison with healthy controls (33). Although Wilk et al. did not observe substantial expression of proinflammatory cytokine genes on circulating monocytes in COVID-19, they did identify severe cases of COVID-19 showing a depletion of CD16^+^ monocytes and a prominent shift toward CD14^+^ monocytes (34). Macrophages are often derived from circulating monocytes and actively implicated in the pathogenesis of a cytokine storm. Activated macrophages could secret an excessive amount of inflammatory cytokines, including IL-1, IL-6, IL-18, and MCP-1 (35). ScRNA-seq analysis reveals an accumulation of classic M1-like and alternative M2-like inflammatory macrophages in patients with severe COVID-19 (36). Compared to the moderate type of COVID-19 patients and healthy controls, macrophages in the lungs of severe cases are more prone to express inflammatory cytokines and chemokines, indicative of a contribution to the local inflammatory environment. Moreover, the M2-like subset is related to tissue repair and fibrosis as well as suppression of antiviral effector T cell responses, eliciting concerns about fibrotic complications and immunodeficiency in COVID-19, respectively. Notably, autopsy reports show that secondary lymphoid tissue–resident CD68^+^ and CD169^+^ macrophages of COVID-19 patients expressed ACE2 and contained the SARS- CoV-2 nucleoprotein (31). In addition, SARS-CoV-2–infected macrophages are triggered to secrete IL-6. Collectively, these findings provide evidence about the pathological role of inappropriate macrophage responses in SARS-CoV-2 infection. Studies show depleted peripheral NK cells in severe cases of COVID-19 in comparison with that in mild cases and healthy controls (33, 34, 37). Furthermore, an exhausted phenotype of peripheral NK cells with a high expression of NKG2A was observed in COVID-19 patients. Notably, scRNA-seq analysis shows that BALF samples in patients with various severities of COVID-19 had a higher percentage of NK cells than those in healthy people (36), which raises the possibility of the trafficking of peripheral NK cells to inflamed lungs. The scanty functional NK cells and exuberant inflammatory monocytes in the peripheral blood may fuel the dissemination of SARS-CoV-2 and the successive serious injury of extrapulmonary organs.

### COVID-19: Adaptive Immune Dysregulation

The activation of the innate immune system and the resultant secretion of proinflammatory cytokines and chemokines subsequently recruit a variety of adaptive immune cells to the inflamed site to prime corresponding adaptive immune responses, including CD8^+^ effector T cell–mediated cytotoxicity, CD4^+^ T cell–dependent cytokine release, and B cell activation–dependent antibody production. Orchestrated innate and adaptive immune responses are capable of clearing the novel coronavirus. In parallel, inflammatory cytokines generated by innate and adaptive immune cells steadily decline (22). Nonetheless, COVID-19 patients with disease progression present with abnormal immune profiles, including sustained high levels of circulating proinflammatory factors (IL-6, IL-1β, IL-2, IL-8, IL-17, IL-18, IL-22, G-CSF, GM-CSF, IP10, MCP1, MIP1α, and TNF-α, etc.) (22, 38–41), known as a “cytokine storm,” as well as lymphopenia (involving reduced numbers of CD4^+^ T cells, CD8^+^ T cells, and NK cells) (22, 40) with the remaining T and NK cells tending to exhibit reduced functional diversity and a more exhausted phenotype (34, 42, 43). As mentioned, SARS-CoV-2 has exploited various strategies to achieve immune evasion. The persistent infection state leads to prolonged immune activation of both the innate and adaptive immune systems. Innate and adaptive immune cells secret large amounts of immune-active molecules to recruit and activate each other (35). With the lack of an efficient negative feedback system, immune cells in severe COVID-19 exert cascade-amplification effects, which lead to collateral tissue damage in the lung and other organs, resulting in cardiovascular, gastrointestinal, renal, and hepatic injuries (44). The cytokine storm is recognized to be a critical pathogenic contributor to the development of ARDS (45). The influx of various immune cells and accumulation of inflammatory mediators damage the alveolus–capillary membrane with consequent fluid accumulation in the lungs leading to difficulty of gas exchange. Moreover, the severe disease course could finally progress into multiple organ dysfunction syndromes, which have been observed in severely infected patients with COVID-19 (46). Furthermore, critical COVID-19 patients are prone to develop coagulation disorders as it is reported that a 31% incidence of thrombotic complications occur in COVID patients admitted to the ICU, including acute pulmonary embolism (PE), deep-vein thrombosis (DVT), and so on (47). The observed hemostatic disorders could be attributed to hyperinflammation-induced endothelial dysfunction (48). In addition, autoimmunity against phospholipids in COVID-19 may be contributory to the development of abnormal coagulation. Half of in-hospital COVID-19 patients had at least one type of serum antiphospholipid antibodies when eight types of antiphospholipid antibodies were measured (49). The autoantibodies to phospholipids not only predispose the endothelium into a prothrombotic state, but they trigger the release of neutrophil extracellular traps (NETs) from neutrophils, which further exacerbates thrombus formation through several mechanisms. Of note, the potently increased serum levels of CXCL9 (chemoattractants of T cells), CXCL16 (chemoattractant of NK cells), C-C motif chemokine 8 (CCL8), CCL2 (recruiting monocytes and/or macrophages), and CXCL8 (majorly attracting neutrophils) were observed in patients infected with SARS-CoV-2 compared with noninfected individuals (19, 22), suggesting a potential contribution of these immune cells in hypercytokinemia.

As mentioned, lymphopenia is a salient feature of SARS-CoV-2 infection. Besides the unproven direct virus-induced lymphocyte disruption and sequestration in the lung lesion (41), lymphocyte apoptosis induced by inflammatory cytokines, such as IL-6 and TNF-α, may also play a pivotal role (50). Notably, evidence shows a dysregulated profile of the adaptive immune system in the severe disease course with relatively high humoral immune and low cellular immune responses, supported by increased amounts of circulating total and effector B cells as well as reduced T effector cells (20, 51). As part of the humoral immune response to SARS-CoV-2, virus-specific IgM and IgG were detected on days 7 and 10 after illness onset, respectively. Severe COVID-19 cases are prone to display earlier and higher titers of IgM and IgG, indicative of a potential correlation of strong antibody response with disease severity (52). Of note, a recent study reveals a specific IgG Fc structure characterized by reduced fucosylation in adult COVID-19 patients (53). The modified Fc structure confers IgG 5- to 10-fold higher affinity to Fc gamma receptor IIIa (FcγRIIIa) in immune cells. As such, antibody-dependent enhancement (ADE) could theoretically potentiate disease through antibody-mediated enhanced viral infection and immune response (54, 55). Currently, the effect of the modified IgG Fc structure on the pathogenesis of COVID-19 remains unclear and needs further clarification. Regarding the cellular immune response, a subgroup of excessively activated and end-differentiated CD8^+^ T cells with diminished function of antigen recognition overwhelmingly dominate in total CD8^+^ T cells (51). Longitudinal analysis of immune profiling of COVID-19 finds elevated plasma levels of IL-17A and IL-22 along with increased IL-17 secretion by CD4^+^ T cells in severe patients, indicative of promotive effects of Th17 cells in COVID-19 progression (22). Moreover, a previous observational study also confirms the pathologic role of Th17 cells in COVID-19 (41). These abnormal adaptive immune dynamics are likely to further fuel inflammation cascade and viral dissemination. Additionally, low expression of human leukocyte antigen-DR (HLA-DR) on CD14 monocytes were detected in COVID-19 patients inversely correlating with a high circulating level of IL-6 (56), suggesting a sepsis-induced immunoparalysis. Moreover, a longitudinal study discovers a massive expansion of T cell–suppressing myeloid-derived suppressor cells in COVID-19 patients, accounting for 90% of total circulating mononuclear cells in severe cases and demonstrating a downward tendency along with recovery (57). The resultant immunodeficient state is likely to predispose COVID-19–infected patients to secondary bacterial and fungal infections in clinical course (3).

## Potential Immunotherapies for COVID-19

### IL-6R Blockades

The strong positive correlation between circulating IL-6 levels and illness severity in hospitalized COVID-19 patients provides the rationale for deployment of IL-6R inhibitors in a selection of infected patients. The most explored reagents that can neutralize IL-6 activity are the humanized monoclonal antibody (mAb) tocilizumab, and the fully human mAb sarilumab, which have previously shown benefits in patients with various rheumatic diseases, such as rheumatoid arthritis (RA), juvenile idiopathic arthritis (JIA), and adult-onset Still’s disease (AOSD) (58). Current results from clinical studies evaluating IL-6R inhibitors in COVID-19 are inconsistent.

The first study includes 21 severe or critical patients treated with tocilizumab with no control group. All 21 patients received routine treatment for a week preceding tocilizumab therapy but exhibited sustained symptom deterioration. After treatment with tocilizumab, patients demonstrated absorbed lung lesions (90.5%), decreased oxygen requirement (75%), normalized lymphocytes (52.6%), and improved clinical conditions (100%) (59) (**Table 2**). Another study analyzed 15 COVID-19 patients with a median age of 73 years, encompassing mild (*n*=2), severe (*n*=6), and critical (*n*=7) types. Ten patients had commodities, and 8 patients received concurrent methylprednisolone treatment. Despite tocilizumab mitigating the elevated CRP levels in all patients rapidly, only one patient achieved clinical improvement, nine were clinically stabilized, and two experienced disease deterioration. It is noteworthy that, instead of decreasing, a perpetuated and marked increase of IL-6 was observed in four patients with low pretreatment plasma IL-6 (16.4–73.6 pg/mL), who eventually succumbed or had disease aggravation (60). In addition, a clinical study from Italy analyzed 21 COVID-19 patients (median age 64 years) with ARDS. All patients were treated with siltuximab at doses ranging from 700 to 1200 mg (median 900 mg) among whom 33% of patients experienced an improved clinical condition, 43% of patients clinically stabilized, and 24% experienced condition deterioration (61). Roumier et al. report 30 selected patients (age <80 years, prior disease duration >5 days) with rapidly worsening (i.e., increase by more than 3 L/min of oxygen flow within the previous 12 h) severe COVID-19–associated pneumonia (62). In comparison with age-, gender-, and disease severity–matched control patients, tocilizumab strikingly reduced mechanical ventilation requirement and risk of subsequent ICU admission. Of note, a trend toward lower mortality, although no statistical difference, was observed in tocilizumab-treated patients. A single-center cohort study tested tocilizumab in patients with COVID-19 requiring mechanical ventilation, and the clinical outcomes of 78 patients who received tocilizumab with 76 well-matched tocilizumab-untreated controls were compared (63). Generally, the use of tocilizumab was associated with improved survival compared with tocilizumab-untreated patients. It is noteworthy that patients receiving tocilizumab treatment in this study were more inclined to develop superinfections than the tocilizumab-untreated controls (54% vs. 26%; *P* < 0.001) although there was no statistical difference in the 28-day case fatality rate between these two groups (22% vs. 15%; *P* = 0.42). Potere et al. report a retrospective case-control study assessing the efficacy and safety of tocilizumab in COVID-19 patients with pneumonia, hyperinflammation, and hypoxemia requiring oxygen therapy (64). Forty eligible patients received tocilizumab treatment in addition to standard supportive care, and another 40 age- and sex-matched patients served as the control group only receiving standard of care. The results show that the application of tocilizumab resulted in good tolerance, improvement in oxygenation, less disease deterioration, and reduced CRP levels in tocilizumab-treated patients compared with the control group. Nevertheless, a case report found no beneficial therapeutic effect of tocilizumab on one severe COVID-19 patient with low pretreatment plasma IL-6 (74.3 pg/mL), who developed viral myocarditis and lymphopenia after receiving tocilizumab treatment (65).

**Table 2:** Study types and characteristics of several clinical studies of immunotherapies.

	Author	Study type	Single or multicenter	Disease severity	Sample size
IL-6R blockades Tocilizumab Sarilumab	Xu et al. (59)	Observational	Multicenter	Severe or critical	21 treated
	Luo et al. (60)	Observational	Single center	Moderate, severe, and critical	15 treated
	Gritti et al. (61)	Observational	Single center	Complicated with ARDS	21 treated
	Roumier et al. (62)	Observational	Single center	Severe (rapidly deteriorating)	30 treated + 29 control
	Somers et al. (63)	Observational	Single center	Severe	78 treated + 76 control
	Potere et al. (64)	Observational	Single center	Severe	40 treated + 40 control
	Radbel et al. (65)	Case series	Single center	Complicated with CRS	2 treated
Convalescent plasma	Duan et al. (66)	Case series	Single center	Severe	10 treated
	Shen et al. (67)	Case series	Single center	Critical (complicated with ARDS)	5 treated
	Zhang et al. (68)	Case series	Single center	Critical	4 treated
	Zeng et al. (69)	Observational	Multicenter	Critical	6 treated + 15 control
	Salazar et al. (70)	Observational	Single center	Severe or critical	25 treated
	Li et al. (71)	RCT	Multicenter	Severe or life-threatening	52 treated + 51 control
	Xia et al. (72)	Observational	Single center	Severe or critical	138 treated + 1430 control
IVIG	Cao et al. (73)	Case series	Single center	Severe	3 treated
	Xie et al. (74)	Observational	Single center	Severe or critical	58 treated
Thymosin alpha1	Liu et al. (75)	Observational	Multicenter	Severe	36 treated + 40 control
	Yu et al. (51)	Observational	Single center	Severe or critical	11 treated + 14 control
Corticosteroids	Ling et al. (76)	Observational	Single center	Not mentioned	5 treated + 61 control
	Zha et al. (77)	Observational	Multicenter	Mild	11 treated + 20 control
	Wang et al. (78)	Observational	Single center	Confirmed COVID-19	73 treated + 42 control
	Wu et al. (79)	Observational	Single center	Complicated with ARDS	50 treated + 34 control
	Yang et al. (80)	Case series	Single center	Complicated with CRS	15 treated
	Wang et al. (81)	Observational	Single center	Severe	26 treated + 20 control
	Liu et al. (82)	Observational	Multicenter	Confirmed COVID-19	40 treated + 97 control
IFNs	Zhou et al. (20)	Observational	Single center	Confirmed COVID-19	53 treated + 24 control
	Hung et al. (83) Wang et al. (84)	RCT Observational	Multicenter Multicenter	Mild to moderate Confirmed COVID-19	86 treated + 41 control 242 treated + 204 control

ARDS, acute respiratory distress syndrome; COVID-19, corona virus disease 2019; CRS, cytokine release syndrome; IFNs, interferons; IL-6R, interleukin 6 receptor; IVIG, intravenous gammaglobulin; RCT, randomized controlled trial.

Generally, many currently available clinical results regarding IL-6 are conflicting and inconsistent, likely due to the differences in clinical trial designs although these data also suggest that disease severity and treatment time points may affect the therapeutic efficacy of IL-6R inhibitors on COVID-19. It should be particularly noted that IL-6 has the dual function of suppressing or prompting viral replication in experimental model systems (85). IL-6 also plays key roles in tissue homeostatic and repair responses (86). The rebound from low pretreatment plasma IL-6 levels in four patients may be a consequence of compensatory synthesis and release of IL-6. As such, the increase of IL-6 to some extent may exert a beneficial effect on infected patients, especially in the later immunodeficient stage of COVID-19. Therefore, we recommend prudent use of IL-6–targeting therapies in concert with antiviral agents in patients with early, rapid, continuous deterioration as well as with clinical and laboratory proof of overactive inflammatory responses (i.e., rapidly aggravating state of breathing, shock, and elevations in IL-6 and CRP). Meanwhile, clinicians should be cautious of a possible IL-6 rebound, successive injuries, and the occurrence of probable infections secondary to IL-6–targeting therapy (87).

### Convalescent Plasma

Convalescent plasma (CP) therapy, as a strategy of passive immunization, has been used in the containment of infectious diseases since the early 20th century (88). CP demonstrated therapeutic benefits in several previous epidemics, including SARS, Middle East respiratory syndrome (MERS), and influenza A H1N1 virus infection (89–91). The protective effect of CP is mainly dependent on the amount and types of antibodies contained in the plasma of convalescent donors. Sufficient neutralizing Abs bind to a specific virus to impede its infectious power directly, thereby suppressing viremia (92). In addition, other nonneutralizing Abs may also confer benefits by exerting immunomodulatory effects. Studies show IgG transferred by plasma possesses anti-inflammatory capability regarding neutralizing cytokines, such as IL-1β and TNF-α (93). Other antibody-mediated pathways, such as complement activation, opsonization, and antibody-dependent cellular cytotoxicity, may also play a favorable role in CP therapy (94). Furthermore, anti-inflammatory cytokines, defensins, and other proteins involved in CP are expected to provide adjuvant therapeutic benefits for severely infected patients (95). In the rampantly spreading COVID-19 pandemic, CP is reevaluated in the absence of specific treatment, and the therapeutic effect of CP is generally encouraging.

Duan et al. report 10 severely ill COVID-19 patients transferred with 200 mL of CP with high neutralizing antibody titers from recently recovered donors (66). The median time from symptom onset to CP transfusion was 16.5 days. After receiving CP transfusion, all patients experienced improved clinical conditions by showing no symptoms or dramatically improved symptoms as well as the recovery of pulmonary lesions. Laboratory parameters are also inclined to improve, evidenced by increased lymphocyte count and decreased CRP levels in CP-treated patients. Notably, the SARS-CoV-2 viral load becomes undetectable in 7 patients receiving CP transfusion. Another uncontrolled case series analyzed 5 critically ill COVID-19 patients with ARDS in which all patients were treated between 10 and 22 days after admission with CP containing SARS-CoV-2 antibodies (titer > 1:1000) and a neutralization titer greater than 40 (67). All patients received antiviral agents and methylprednisolone before CP therapy. After CP treatment, four out of five patients displayed clinical improvements, including normalized body temperature, elevated PaO_2_/FiO_2_, and resolved ARDS. The decrement of viral loads to an undetectable level was also seen within 12 days after the transfusion. Of the same five patients, three have been discharged, and two have been clinically stabilized. Zhang et al. retrospectively describe four critically ill patients who were transfused with different amounts of CP (200−2400 ml) varying between 11 and 28 days after admission (68). Among the four patients, the time from transfusion to negative SARS-CoV-2 RNA tests ranged from 3 to 22 days. All the patients recovered from the SARS-CoV-2 infection, including three discharged from the hospital and one transferred to unfenced ICU. Zeng et al. evaluate the efficacy of CP in 6 critically ill COVID-19 patients with respiratory failure (69). All infected patients received CP transfusion at a median of 21.5 days after the first detection of viral shedding. Although SARS-CoV-2 turned negative in all subjects by 3 days after initiating CP therapy, five out of six patients are deceased. In a study of 25 patients with severe and/or life-threatening COVID-19 disease, all patients received one 300-mL dose of CP administrated at a median time of 10 days with 1 patient receiving a second transfusion 6 days after the initial transfusion (70). Most patients received concomitant anti-inflammatory agents and investigational antiviral treatments. On day 7 post-transfusion, 9 patients (36%) had improved clinical status, and 7 patients were discharged. By 14 days post-transfusion, 19 patients (76%) were clinically improved or discharged. Specifically, no adverse events pertinent to plasma transfusion were observed. In the newly reported randomized clinical trial of CP therapy for COVID-19 conducted in China, Li et al. tested the role of CP in severe (respiratory distress and/or hypoxemia) or life-threatening (shock, organ failure, or requiring mechanical ventilation) COVID-19 patients with 52 randomized patients concurrently receiving standard care serving as the treatment group and another 51 randomized patients who received standard supportive care alone serving as controls (71). This randomized controlled trial involving 103 patients was originally planned to recruit 200 patients; however, the trial was terminated prematurely. Consequently, the clinical study was underpowered to some extent. In addition, there are no statistically significant differences pertaining to clinical improvement, discharge rate, and 28-day mortality rate between the CP-treated and control groups. Nonetheless, the report indeed indicates hopeful signals of CP therapy in COVID-19 patients, especially in severe types. The clinical improvement at 28 days occurred in 27 patients (51.9%) in the CP group vs. 22 patients (43.1%) in the control group (hazard ratio (HR), 1.40 [95% CI, 0.79−2.49]; *P* = 0.26). Negative SARS-CoV-2 conversion at 72 h occurred in 87.2% of the CP group vs. 37.5% of the control group (OR, 11.39 [95% CI, 3.91−33.18]; *P* < 0.001). According to disease severity–oriented stratified analysis, in severe cases, the clinical improvement within 28 days occurred in 91.3% (21/23) of the CP group vs. 68.2% (15/22) of the control group (HR, 2.15 [95% CI, 1.07−4.32]; *P* = 0.03) although, in subjects with a life-threatening disease, it occurred in 20.7% (6/29) of the convalescent plasma group vs. 24.1% (7/29) of the control group (HR, 0.88 [95% CI, 0.30−2.63]; *P* = 0.83). In terms of the primary outcome, in severely ill patients, the time to clinical improvement within 28 days was 4.94 days shorter in the CP group relative to the control group (95% CI, -9.33−-0.54 days). Specifically, only 2 patients in the CP group experienced adverse events related to CP transfusion.

A single-center study reported on 1568 patients infected with severe or critical COVID-19 disease in Wuhan, of whom 138 patients received 200−1200 ml CP transfusion depending on different clinical status and body weight, and another 1430 patients only received standard of care (72). CP treatment reduced the fatality rate by approximately 50% in the standard-treatment group (2.2% vs. 4.1%). Encouragingly, CP therapy was associated with an increased percentage of lymphocytes and decreased percentage of neutrophils and CRP levels. Except for the decrease in total bilirubin, no serious adverse event associated with CP therapy was observed.

These six studies testing the therapeutic effectiveness of CP in COVID-19 are with limited controls and a relatively small sample size, which make them hard to be conclusive. The clinical trial conducted by Li et al. used a randomized trial design and recruited a relative larger sample size; however, it failed to acquire a statistically significant clinical improvement, possibly due to the early termination (71). Nevertheless, the therapeutic effectiveness of CP for COVID-19 reflected by these clinical trials are fairly hopeful, considering the majority of enrolled patients are in the advanced disease course with severe viremia. Moreover, it is generally recognized that viremia maximizes in the first week of infection in most viral illnesses; thus, an early CP infusion is likely to achieve the best therapeutic effect. However, a substantial number of infected patients in these studies failed to receive CP infusion until their disease progressed into severe or critical types. The protective effect of CP depends on the amount and types of antibodies. The possible mechanisms include viral neutralization and antibody-dependent cellular cytotoxicity (94). Collectively, according to the currently available clinical data, CP was well tolerated in COVID-19 patients and has an acceptable safety profile.

### Intravenous Gamma Globulin

Intravenous gamma globulin (IVIG) is a purified blood product containing polyclonal immunoglobulin G prepared from thousands of healthy donors. It has long been used for treating a range of autoimmune and chronic inflammatory diseases, such as immune thrombocytopenic purpura (ITP), Kawasaki disease (KD), and primary immunodeficiency disease (96). Furthermore, in the past pandemic of influenza A H1N1 virus and the epidemics of SARS and MERS, application of IVIG alone or combined with other therapies is associated with the reduction of viral burden and various improvements in clinical outcomes (97–99). Currently, the experience from the application of IVIG in the treatment of SARS-CoV-2 infection is very limited.

Cao et al. report a case series of 3 patients with COVID-19 receiving a high dose of IVIG (0.3–0.5 g per kg weight per day) for 5 days (73). All 3 patients experienced varied elevations of inflammatory biomarkers (i.e., hsCRP, ESR). Furthermore, 2 patients exhibited lymphocytopenia, 1 patient displayed mild thrombocytopenia, and all 3 patients deteriorated into severe types before IVIG therapy. After infusion with IVIG, they all had normalized temperature within 2 days after treatment and alleviated respiratory symptoms within 5 days. None of them was reported with any adverse events. However, the effects of concurrent treatments, including antiviral agents and steroids, confounded the exact therapeutic effectiveness of IVIG. A retrospective study by Xie et al. explores the role of IVIG in severe and critically ill patients (74). The study included 58 severely or critically infected patients. All patients received supportive care and antiviral treatments. Once the absolute lymphocyte count fell below 0.5 × 10^9^/L, they commenced with IVIG infusion (20 g/day) and correction for hypoalbuminemia. The study shows that the use of IVIG within 48 h could significantly reduce the 28-day mortality rate, ventilator requirement, and hospital and ICU stay although an earlier use of IVIG within 24 h after admission did not make any statistical difference in terms of the 14- or 28-day mortality rates, suggesting the importance of choosing the appropriate initiation time for IVIG therapy.

The anti-inflammatory and immune-modulatory properties of IVIG provide the rationale for its use in patients with SARS-CoV-2 infection. The possible mechanisms facilitating restoration of immune homeostasis through IVIG include blocking multiple proinflammatory cytokines by autoreactive antibodies, blocking activating FcγR on innate immune effector cells, suppressing pathogenic Th1 and Th17 subsets, and expanding regulatory T cells (100, 101). Additionally, a mouse model study shows that IVIG could reduce gut inflammation; the beneficial effect is related to the suppression of IL-6 and the enhancement of IL-10 in the gut (102). A recent case report shows that a severe COVID-19 patient with sustained diarrhea recovered after the treatment of intensive plasma exchange and IVIG (103). The amelioration of respiratory symptoms and diarrhea is, at the least, attributable to the immuno-regulatory effect of IVIG. However, a clear demonstration of effectiveness and safety of IVIG requires well-controlled clinical studies.

### Thymosin Alpha1

Thymosin alpha1 (Tα1) is a kind of endogenous polypeptide first isolated from the thymic tissue, and it has been applied in multiple debilitating diseases ranging from cancers and autoimmune disorders (104, 105) to viral infectious diseases (106). Tα1 is demonstrated to have immune-regulatory effects, including enhancing the production of T cells, the level of HDR, and anti-inflammatory cytokine IL-10 while decreasing proinflammatory cytokine TNF-α (107, 108). Considering the ubiquity of lymphocytopenia in patients infected with COVID-19 and its deleterious effects, the adjuvant use of Tα1 might yield some therapeutic benefits through prompting T cell production.

Liu et al. retrospectively report on 76 severe or critical COVID-19 patients with 36 patients receiving treatment of Tα1 and 40 nontreated patients serving as a control group (75). Tα1-treated patients were given subcutaneous injections of Tα1 (10 mg once per day) for at least 7 consecutive days. In comparison with the nontreated patients, Tα1 significantly reduced the mortality rate of infected patients, and effectively restored T cell numbers in patients with severe lymphocytopenia (CD8^+^ T cells < 400/μL, CD4^+^ T cells < 650/μL). Furthermore, it is noteworthy that patients older than 60 years or with underlying comorbidities of hypertension or cardiovascular diseases, also exhibited an enhancement of CD8^+^ T and CD4^+^ T cells. In additio, Tα1 refrains T cells from exhaustion mirrored by the decreased expression of exhausted markers involving programmed death-1 (PD-1) and T cell immunoglobulin and mucin domain protein 3 (Tim-3) on CD8^+^ T cells post Tα1 therapy. Tα1 exerts its promotional effect on the restoration of CD8^+^ T and CD4^+^ T cells through boosting thymus output. No adverse event related to Tα1 was reported. Yu et al. performed single-cell transcriptomic analysis of peripheral lymphocytes from 10 patients at different stages of the disease and found abnormal dynamics of adaptive immune cells (51). Prothymosin alpha (PTMA), the proprotein of Tα1, was significantly expressed in a subset of stem-like *CD8^+^ T* memory cells with a highly proliferating feature and low expressed on a cluster of dysfunctional and overactivated *CD8^+^ T* cells. In subsequent *in vitro* tests, Tα1 dramatically increased T cell numbers on days 6 and 9 compared with the untreated group. Furthermore, Tα1 reduced the production of IFNγ and TNFα although not statistically significantly.

In general, the reports regarding the effectiveness of Tα1 in COVID-19 are pretty limited although Tα1 was clinically applied as an adjuvant therapy in severely or critically infected patients at the beginning of the SARS-CoV-2 outbreak. To date, the preliminary results of Tα1 for treating COVID-19 revealed by the only two available clinical studies are greatly encouraging. The equilibrium of the adaptive immune system is of paramount importance. Sufficient capability to eradicate viral invasion and restrain excessive secretion of inflammatory cytokines constitute an efficient adaptive immune response to SARS-CoV-2. As shown by Yu et al., Tα1 could restore T cell numbers, which are essential for viral clearance, concomitant with decreased production of IFNγ and TNF-α in the case of formation of a deleterious inflammation cascade (51). Hence, Tα1 is a promising treatment option for COVID-19 patients, especially for those who manifest severe lymphocytopenia.

### Corticosteroids

In clinical settings, corticosteroids have been empirically administrated in COVID-19 patients, especially in critical cases with rapid disease deterioration (38). Corticosteroids are a major medication of immunomodulatory therapy functioning by potent anti-inflammatory and antifibrotic capabilities, and they have been widely used as an auxiliary therapy in the treatment of ARDS; sepsis; and viral infectious diseases, such as SARS, MERS, and influenza (109–111). However, clinical results on the role of corticosteroids are discrepant, and the controversial situation still persists in SARS-CoV-2 infection.

A retrospective study involved 113 inpatients, 56.6% of whom received relatively low corticosteroid dosing (0.5−1.0 mg methylprednisolone/kg body weight) (112). Analysis indicates that corticosteroid treatment is related to prolonged SARS-CoV-2 RNA shedding but does not serve as an independent risk factor. Similarly, in another study, 5 patients treated with corticosteroids displayed longer duration of viral RNA detection compared with the non-glucocorticoid treatment group, indicative of delayed virus clearance induced by corticosteroid treatment (76). However, according to a cohort study including 120 noncritical inpatients (54/120 receiving corticosteroid therapy), corticosteroid treatment is unrelated to prolonged SARS-CoV-2 RNA shedding (113). The result is in parallel with an observational study showing no association between corticosteroid treatment and virus clearance time (77). In this report, 11 of 31 mild COVID-19 patients were administrated with corticosteroids (40 mg methylprednisolone once or twice per day) within 24 h of admission for a median 5 days. In addition to unaffected virologic outcomes, corticosteroid treatment did not influence the length of hospital stay and duration of symptoms in mild cases. According to a cohort study, severe COVID-19 infected patients were more inclined to receive corticosteroid treatment compared with nonsevere patients (114). Furthermore, high-dose corticosteroid use (maximum dose ≥1 mg/kg/d prednisone) was related to a high risk of death in severe cases. Another observational study did not support the usage of corticosteroids as corticosteroid treatment was significantly associated with adverse outcomes of either mortality or ICU admission (78). After adjusting for confounding factors on outcomes, although no statistical significance was observed, a 2.155-fold increase in risk in either mortality or ICU admission was observed in corticosteroid-treated patients in comparison with non-corticosteroid-treated patients.

Several clinical studies also report beneficial effects of corticosteroid treatment on COVID-19 patients. A cohort study including 84 COVID-19 patients with ARDS indicates that corticosteroid therapy may lead to significant mortality reduction compared to non-corticosteroid therapy (46.0% vs. 61.8%) in seriously ill COVID-19 patients with ARDS (79). Additionally, a case series report involving 15 ICU patients showed corticosteroid therapy was related to a reduced requirement for vasopressor, improved oxygenation, and decreased CRP levels in critical patients (80). Of note, the enrolled 15 patients all developed respiratory failure with progressing increased inflammatory markers, suggesting the potential benefit of corticosteroids in critical types with hyperinflammation. Moreover, the application of corticosteroids in an early, low-dose, and short-term strategy have demonstrated favorable effects on 26 patients with severe COVID-19 pneumonia (81). In this study, methylprednisolone was intravenously administrated at a dosage of 1−2 mg/kg/d for 5−7 days. Compared with those without methylprednisolone administration, patients receiving methylprednisolone treatment presented with more rapid temperature normalization, faster oxygenation improvement, and better lung radiography. Nonetheless, in another observational study including 137 patients, small dosage of methylprednisolone (30–80  mg/day) failed to rescue a cluster of COVID-19 patients with short-term disease progression (82). Forty patients administrated with methylprednisolone did not show a shortened disease course or an improved clinical outcome, and some even displayed aggravated lung images after methylprednisolone therapy.

British researchers have announced surprising results in an evaluation of corticosteroids for treating COVID-19 in the recovery trial (115). In this recently published, large-scale, controlled, open-label trial, 2104 patients were assigned to receive oral or intravenous dexamethasone treatment (at a dose of 6 mg once daily) for up to 10 days, and 4321 patients received usual care. Generally, application of dexamethasone in hospitalized COVID-19 patients led to lower 28-day mortality than the usual care (22.9% vs. 25.7%; *P* < 0.001). Furthermore, clinical benefits of dexamethasone were closely associated with respiratory support. Receipt of dexamethasone was associated with lower mortality rates in comparison with the usual care group among patients requiring invasive mechanical ventilation (29.3% vs. 41.4%) and in those receiving supplemental oxygen in other ways (23.3% vs. 26.2%). Nonetheless, there was a trend showing a seemingly harmful effect of dexamethasone treatment among patients without respiratory support (17.8% vs. 14.0%). Importantly, dexamethasone seemed to confer a clear benefit in patients treated for more than 7 days after symptom onset when the lung damage commonly developed. Additionally, dexamethasone treatment also demonstrated more therapeutic benefits than usual care in terms of duration of hospital stay (median, 12 vs. 13 days), probability of discharge alive within 28 days, and risk of progression to invasive mechanical ventilation.

At present, clinical observational studies evaluating the efficacy and safety of corticosteroids in COVID-19 are highly contested and limited by their small sample sizes as well as nonrandomized nature. Therefore, a concrete conclusion about corticosteroid application in the treatment of SARS-CoV-2 infection still cannot be made. According to the aforementioned studies, corticosteroid administration was more common in severe and critical patients in clinical practice; thus, it is more likely that disease severity, rather than the use of corticosteroids, predisposes COVID-19 patients to a high risk of death. In addition, the therapeutic effects of corticosteroids are seemingly affected by the heterogeneity of dosing regimens, duration, and initiation timing of therapy. Encouragingly, corticosteroid therapy has demonstrated therapeutic benefits in COVID-19 patients requiring respiratory support (115) and in a subset of critical patients with ARDS (79), which indicates rational and prudent corticosteroid usage might yield favorable outcomes in patients experiencing respiratory disorders. Given the intertwined hyperinflammatory and immunocompromised stages in the COVID-19 disease course, a short-term and low-dose application of corticosteroids promoting the formation of a disastrous cytokine storm is likely to rescue seriously ill patients from disease aggravation. Considering potential adverse events elicited by corticosteroid therapy, such as hyperglycemia, secondary infection, and osteonecrosis (111, 116), the low-dose administration of corticosteroids for a short duration is needed with simultaneously close monitoring.

### IFNs

IFNs are critical components of the immediate antiviral response. IFNs are classified into three classes, comprising a plethora of type-I IFNs (IFN-α, IFN-β, IFN-ϵ, IFN-κ, IFN-ω in humans), type-II species (IFN-γ), and type-III IFNs (IFN-λ). IFNs could be distinguished by the surface receptors to which they bind. Type-I IFNs bind to IFN-α/β receptors (IFNAR), which are widely distributed in body cells; type-II IFNs bind to the IFN-γ receptor (IFNGR); and type-III IFNs signal through type-III IFN receptors (IFNLR) only expressed on epithelial cells and certain myeloid cells, including neutrophils and human B cells (117). All IFNs are considered to have antiviral effects (118). As mentioned, early and sufficient secretion of type-I IFNs is recognized as beneficial for the control of SARS-CoV-2. Concordantly, Bastard et al. report that individuals who had neutralizing autoantibodies (auto-Abs) to type-I IFNs are more susceptible to develop critical types of COVID-19 once infected by SARS-CoV-2 as the auto-Abs could weaken the ability of type-I IFNs to achieve viral clearance (49).

Of note, the majority of patients (95 of the 101) with auto-Abs are men, which may explain why being male is a risk factor for life-threatening COVID-19.

In previous *in vivo* and *in vitro* studies of viral infectious diseases, type-I IFNs alone or in combination with other antiviral agents have displayed promising efficacy; however, negative results also exist. In a marmoset model study of MERS, the combination treatment of IFN-β1b with lopinavir/ritonavir reduced viral load and lung abnormalities (119). Similarly, the prophylactic administration of IFN-α2b was reported to protect type-1 pneumocytes against SARS coronavirus infection and reduce viral replication in macaques (120). Clinical usage of IFN-a combined with corticosteroids led to better outcomes, including improved oxygen saturation and rapid resolution of lung lesions (121). The application of IFN-α2a combined with ribavirin in MERS patients delayed mortality, whereas it failed to maintain efficacy with a late initiation (122). In another cohort study, the IFN-ribavirin combination was not significantly associated with improved outcomes in patients with MERS (89). Low concentrations of type-I IFNs in severely ill patients with COVID-19 are recognized as a prognostic factor for poor outcomes (20). Thus, it is reasonable to consider adding type-I IFNs to the treatment of COVID-19. Indeed, type-I IFNs and other antiviral therapies have already demonstrated some therapeutic benefits in COVID-19. In a retrospective study of 77 COVID-19 patients, treatment with IFN-α2b with or without arbidol substantially reduced the duration of viral shedding and inflammatory markers, including IL-6 and CRP (20). Furthermore, according to a very recently reported phase II clinical trial, in comparison with monotherapy of lopinavir-ritonavir, a triple combination therapy of IFN-β1b, lopinavir-ritonavir, and ribavirin in mild-to-moderate COVID-19 patients was associated with the shortened duration of viral shedding, reduced length of hospital stay, and improved clinical symptoms (83). Also, a retrospective multicenter cohort study compared patients who received IFN therapies with those who did not receive IFNs and found association of early IFN-a2b administration (<5 days after admission) with a significantly reduced mortality rate in severe-to-critical cases of COVID-19 although late IFN use was associated with prolonged hospital stay and slower improvement of CT scan compared with non-IFN therapy (84).

IFN-γ has been investigated in various allergic diseases with relatively good safety profiles (123). In addition to antiviral effects, IFN-γ is also shown to have a potent immunomodulatory property (124). In a SARS-CoV infected mouse model, intraperitoneal IFN-γ injection effectively protected adult mice against lethal injury (125). Bellomi et al. also report IFN-γ and IFN-β could synergistically counter against SARS-CoV infection on Vero cells (126). Importantly, IFN-γ in concert with IL-4 is shown to downregulate the expression of ACE2 on SARS-CoV-infected cells *in vitro* (127), suggestive of its potentially inhibitory activity on SARS-CoV infection.

IFN-λ has recently obtained much attention and serves as a promising treatment option for COVID-19 by virtue of its distinctive mode of action. Because of the restricted expression of IFNLR, IFN-λ is proposed to exert sustained guarding effects on epithelial cells without a deleterious systemic inflammatory effect, which may be a great concern for IFN-I application. Of note, in a newly developed mouse model of SARS-CoV-2 infection, both prophylactic and therapeutic administration of IFN-λ inhibited viral replication in mice (128). However, a recent study investigated the roles of type-III IFNs on lung physiology and surprisingly found that IFN-λ could contribute to the disruption of the lung barrier and increase the risk of lethal bacterial superinfections (129). Likewise, another study indicated potential harm of IFN-λ for pulmonary epithelia, which also reported that the exacerbated and prolonged production of IFNs, including IFN-α/β and IFN-λ, hampered lung epithelial proliferation. IFN-λ shows the most potent deleterious effect (130). Additionally, the sustained and highly elevated plasma levels of IFN-α and IFN-λ were observed in patients who died of SARS-CoV-2 infection (22). Thus, we should stay cautious about the application of IFNs in case of resultant adverse events.

Once antiviral therapies armed by IFNs are considered, the initiation time of IFN therapy is of utmost importance. Early administration preceding the peak of viremia or prophylactic treatment may confer maximal protection without substantial pathology. In addition, regarding possible adverse events, close scrutiny of hallmarks in inflammation and lung function is necessary.

### Vaccine

Despite several immunotherapies demonstrating their therapeutic benefits in the clinical context of controlling COVID-19, there is an urgent need to develop effective vaccines specific against SARS-CoV-2. An effective vaccine could confer individuals the ability to develop immunity to SARS-CoV-2 before being exposed to the virus. Currently, companies and institutions worldwide have taken the initiative of developing vaccines against COVID-19 based on various molecular platforms, such as inactivated virus, live attenuated virus, viral protein subunit, viral nucleic acid, peptide, viral vector (replicating and nonreplicating), and virus-like particle (131). As of early December, there are 13 vaccines that reached phase 3 efficacy trials, of which, mRNA-1273 encodes stabilized prefusion S proteins of SARS-CoV-2 and is embedded in lipid nanoparticles (132). Solicited adverse effects include fever, fatigue, headache, muscle ache, and pain at the injection site (132). Delivery of the antigen into human cells could stimulate the body to produce virus-specific T and B cells. Once the SARS-CoV-2 invasion in encountered in the future, memory T cells rapidly respond to SARS-CoV-2, and memory B cells produce neutralizing antibodies against the virus, thus protecting healthy individuals from COVID-19. Ad5-nCoV is a nonreplicating viral vector vaccine that uses replication-defective adenovirus type 5 as a vector to express S protein, the adverse effects of which are generally moderate, including fever, pain, and fatigue (133). AZD1222 (ChAdOx1-S) is a nonreplicating viral vector vaccine expressing the SARS-CoV-2 S protein with no severe adverse effects (134), and NVX-CoV2373 is a SARS-CoV-2 recombinant spike protein nanoparticle with adjuvant, the reported adverse events of which were mild (135). Despite the vaccines under development varying in types and mechanisms of action, they are all aimed to give instructions on how to build robust immunity to fight against SARS-CoV-2.

## Concluding Remarks

**Figure 1:**
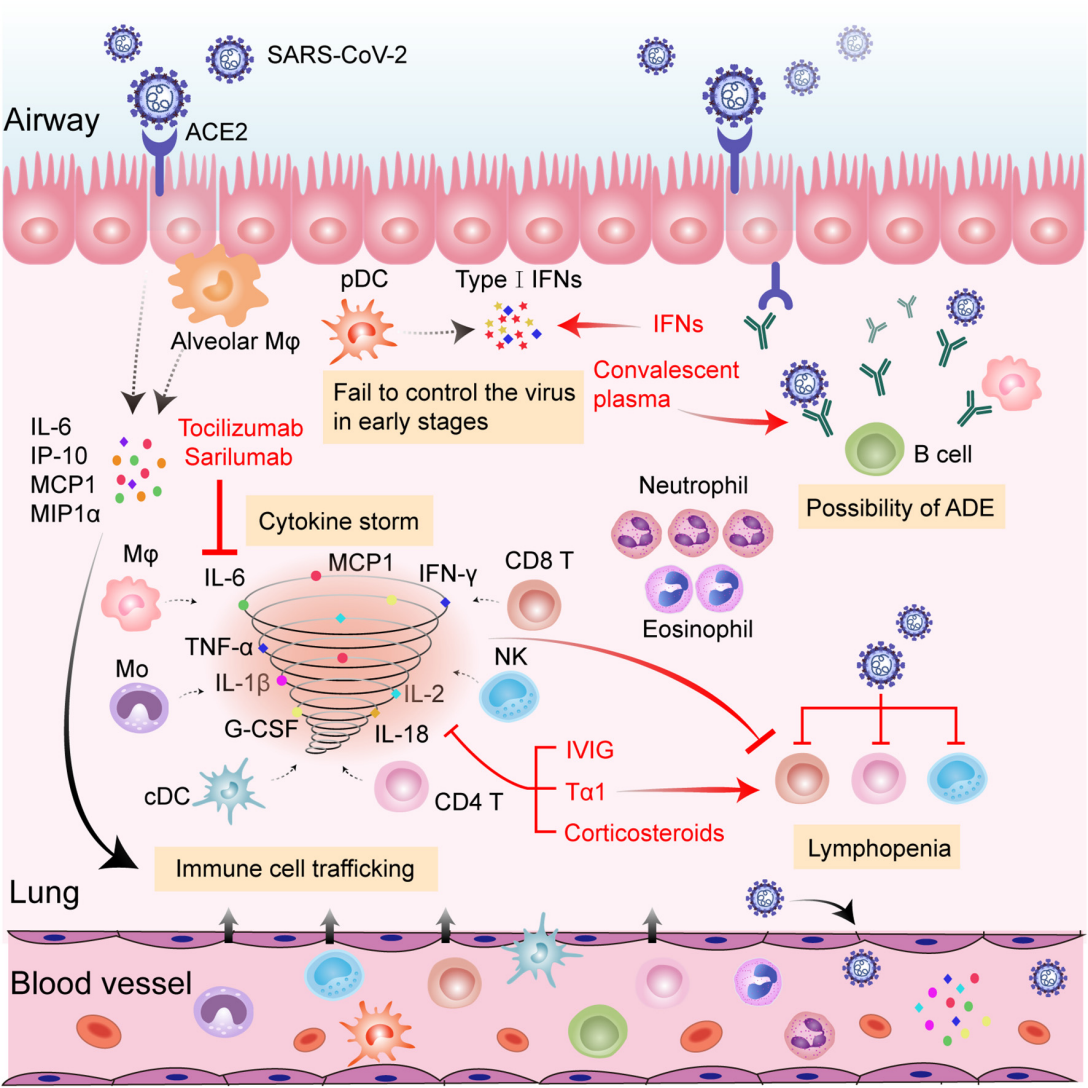
Hypothesized immunopathology of severe SARS-CoV-2 infection and possible therapeutic targets of anti-COVID-19 immunotherapies. SARS-CoV-2 infects pulmonary epithelial cells through binding to ACE2. Viral infection and replication destroy infected lung cells, inducing the release of numerous cytokines and chemokines (i.e., IL-6, IP-10, MCP1, and MIPIα) from neighboring epithelial cells and alveolar Mφ. The released proteins recruit a variety of innate and adaptive immune cells to the inflamed site among which, pDCs are the main source of type-I IFNs. The failure of type-I IFNs to successfully control SARS-CoV-2 in the early stages fuels the viral dissemination to other organs and tissues. Monocytes, Mφ, cDCs, NK cells, CD4^+^ T cells, and CD8^+^ T cells further secrete inflammatory cytokines (including IL-1β, IL-2, IL-6, IL-18, TNF-α, G-CSF, IFN-γ, etc.) and promote the cascade of inflammatory processes. Amounts of neutrophils and eosinophils are attracted to the site of infection and may cause damage to the lungs. Virus-induced lymphocyte disruption and sequestration in the lung lesion, along with inflammatory cytokine-induced lymphocyte apoptosis, may result in lymphopenia in the circulation. Non-neutralizing antibodies produced by B cells may enhance viral infection and potentiate immune responses through ADE. Tocilizumab and sarilumab could neutralize IL-6 activity, thus reducing inflammation. CP confers benefits by providing protective antibodies from recovered patients. IVIG, Tα1, and corticosteroids could alleviate deleterious inflammatory responses. Tα1 could also prompt T cell production. Addition of IFNs could confer antiviral effects. Abbreviations: ACE2, angiotensin-converting enzyme 2; ADE, antibody-dependent enhancement; cDC, conventional dendritic cells; G-CSF, granulocyte-colony stimulating factor; IFN-γ, interferon gamma; IL, interleukin; IP-10, interferon-inducible protein-10; IVIG, intravenous gamma globulin; MCP-1, monocyte chemotactic protein 1; MIPIα, macrophage inflammatory protein-1α; Mo, monocyte; Mφ, macrophage; NLR, neutrophil-to-lymphocyte; NK, natural killer cell; pDC, plasmacytoid dendritic cells; SARS-CoV-2, severe acute respiratory syndrome coronavirus 2; Tα1, Thymosin alpha1; TNF-α, tumor necrosis factor alpha.

The rapid diffusion of COVID-19 has posed a huge threat to public health and the world economy. The continued occurrence and refraction of severe and critical cases further overwhelms the intensive care system. Although our understanding of the immune pathogenesis of COVID-19 is still very limited, it is generally recognized that a disordered immune response is the determinant of the severe types. Hallmarks of hyperinflammation in concert with reduced and dysfunctional lymphocytes are observed in a cluster of severe or critical patients, contributing to ARDS and multiorgan failures. Moderately enhanced cytokines and effectual adaptive immune cells are equally important to successful viral elimination and homeostasis restoration. Thus, exploring a balance between anti-inflammation and maintenance of immune function is vital when considering the initiation of immunotherapies. Protection from excessive inflammation in organs should not be at the expense of immunity. In addition, individually tailored immunotherapy initiated at the right time window could yield maximum therapeutic benefits (**Figure 1**). In the very early stage of COVID-19, the application of antiviral agents with Tα1 is recommended in case of a vicious inflammatory cascade to reduce the viral burden and strengthen immunity. For a subset of rapidly deteriorating infected patients with hallmarks of uncontrolled proinflammatory responses, anti-inflammatory treatments, such as cytokine-targeted therapies, might be beneficial by suppressing hyperinflammation-induced injuries. Meanwhile, close monitoring of adaptive immune function is indispensable. Further immune-related studies about COVID-19 are necessary for guiding rapid disease stratification and well-orchestrated immunotherapies.

## Data Availability Statement

The original contributions presented in the study are included in the article/supplementary material, further inquiries can be directed to the corresponding authors.

## Author Contributions

BX and JZ performed the study design, data collection, data analysis, data interpretation, and writing. YL performed the data collection and data analysis. YS and SY performed the study design, data interpretation, and writing. All authors contributed to the article and approved the submitted version.

## Conflict of Interest

The authors declare that the research was conducted in the absence of any commercial or financial relationships that could be construed as a potential conflict of interest.
